# Comparison of Diagnostic Tools for the Detection of *Dirofilaria immitis* Infection in Dogs

**DOI:** 10.3390/pathogens9060499

**Published:** 2020-06-22

**Authors:** Rossella Panarese, Roberta Iatta, Jairo Alfonso Mendoza-Roldan, Donald Szlosek, Jennifer Braff, Joe Liu, Frédéric Beugnet, Filipe Dantas-Torres, Melissa J. Beall, Domenico Otranto

**Affiliations:** 1 Department of Veterinary Medicine, University of Bari, 70010 Valenzano, Bari, Italy; rossella.panarese@uniba.it (R.P.); roberta.iatta@uniba.it (R.I.); jairo.mendozaroldan@uniba.it (J.A.M.-R.); 2 IDEXX Laboratories, Inc., Westbrook, ME 04092, USA; Donald-Szlosek@idexx.com (D.S.); Jennifer-Braff@idexx.com (J.B.); Joe-Liu@idexx.com (J.L.); Melissa-Beall@idexx.com (M.J.B.); 3 Boehringer Ingelheim Animal Health, 69007 Lyon, France; Frederic.BEUGNET@boehringer-ingelheim.com; 4 Aggeu Magalhães Institute, Oswaldo Cruz Foundation, 50740-465 Recife, Brazil; filipe.dantas@cpqam.fiocruz.br; 5 Faculty of Veterinary Sciences, Bu-Ali Sina University, Hamedan 6516738695, Iran

**Keywords:** *Dirofilaria immitis*, modified Knott’s test, ELISA, immune complex dissociation, serological assays

## Abstract

In the last two decades, reports of canine heartworm (HW) infection have increased even in non-endemic areas, with a large variability in prevalence data due to the diagnostic strategy employed. This study evaluated the relative performance of two microtiter plate ELISA methods for the detection of HW antigen in determining the occurrence of *Dirofilaria immitis* in a dog population previously tested by the modified Knott’s test and SNAP 4Dx Plus test. The prevalence of this infection in the sheltered dog population (n = 363) from a high-risk area for HW infection was 44.4% according to the modified Knott’s test and 58.1% according to a point-of-care antigen ELISA. All serum samples were then evaluated by a microtiter plate ELISA test performed with and without immune complex dissociation (ICD). The prevalence increased from 56.5% to 79.6% following ICD, indicating a high proportion of samples with immune complexing. Comparing these results to that of the modified Knott’s test, the samples negative for microfilariae (mfs) and those positive only for *D. repens* mfs demonstrated the greatest increase in the proportion of positive results for *D. immitis* by ELISA following ICD. While the ICD method is not recommended for routine screening, it may be a valuable secondary strategy for identifying HW infections in dogs.

## 1. Introduction

*Dirofilaria immitis* and *Dirofilaria repens* are widespread mosquito-borne filarial worms that may infect and cause mild to severe diseases in a vast range of mammals, including dogs, cats and humans [1,2,3]. *Dirofilaria immitis* is the causative agent of heartworm disease (HWD), while *D. repens* is that of subcutaneous dirofilariosis (SCD) in dogs. *Dirofilaria* spp. infections in humans are oftentimes underestimated or misdiagnosed, but symptomatic cases are usually detected in areas with a high prevalence of infection in dogs [4,5]. In fact, the risk of *Dirofilaria* spp. infections in humans is strongly linked to the presence of infected dogs and competent mosquito vectors [6,7].

In the last two decades, reports of clinical cases of heartworm (HW) infection in dogs have increased even in non-endemic areas with new endemic foci detected in the Mediterranean region [8,9,10]. However, several studies have recorded a large variability in prevalence data [11,12,13], which depends on epidemiological factors (e.g., presence and abundance of competent vectors and absence of chemoprophylaxis treatments) as well as on the diagnostic methods employed. Indeed, the frequency of *Dirofilaria* spp. infections in dogs may differ according to the diagnostic method used, as demonstrated in Slovakia, where 36% and 64% of the dogs were found positive using the modified Knott’s method and heartworm (HW) antigen test, respectively [12]. In fact, HW antigen tests are also able to detect occult infections characterized by amicrofilaremia.

*Dirofilaria immitis* infection in dogs is usually diagnosed by parasitological and serological assays, and eventually confirmed by molecular analyses. The modified Knott’s test is the most popular parasitological method among concentration tests (e.g., acetone, 5% Tween 20 solution, distilled water and 1% or 0.1% SDS) [14,15], being based on the detection and identification of microfilariae (mfs) of *Dirofilaria* spp. in blood samples. However, the results of the modified Knott’s test may be impaired by occult infections as has been observed in up to 67% of dogs positive for *D. immitis* adults [16].

Serological tests (i.e., enzyme-linked immunosorbent assay (ELISA) and immunochromatographic tests (ICT)) for the detection of somatic and female antigens of *D. immitis* adults are available for point-of care (POC) testing or for reference diagnostic laboratories [1,12,17,18]. The antigen tests are highly specific to *D. immitis*. Nevertheless, cross-reactions may occur with antigens of other nematodes (e.g., *D. repens*, *Angiostrongylus vasorum* and *Spirocerca lupi*). These tests are also highly sensitive, particularly when samples are subjected to immune complex dissociation (ICD) methods prior to testing [19,20]. Indeed, the preheating method has been shown to improve antigen detection in both experimental and natural *D. immitis* infections by releasing HW antigen that is bound to host antibodies [21,22,23]. From this perspective, the aims of this study were to evaluate the relative performance of two different microtiter plate ELISA methods for the detection of HW antigen in a sheltered dog population previously tested by a modified Knott’s test in order to evaluate the infection prevalence in this population using combinations of different methods (i.e., modified Knott’s test plus HW antigen tests), and to determine the effect of preheating on HW antigen detection.

## 2. Results

The overall prevalence of *Dirofilaria* spp. infection was 49.3% (179/363) according to the modified Knott’s test, with 44.4% (161/363) positive for *D. immitis*, 7.2% (26/363) for *D. repens* and one positive for a *Dirofilaria* sp. that could not be identified morphologically. Nine cases (already included in their respective totals above) were co-infected by *D. immitis* and *D. repens* (2.5%, 9/363). No other filarial species (e.g., *Acanthocheilonema* spp.) were detected in the canine blood samples analyzed. Table 1 shows the results of the HW antigen detection by the SNAP® 4Dx® Plus test and the microtiter plate ELISA with and without preheating of the samples scored negative on the modified Knott’s test (n = 184).

The proportion of *D. immitis*-positive samples increased to 58.1% (211/363) when Knott’s positive samples were combined with those positive by the SNAP 4Dx Plus test. Out of 134 negative samples by SNAP 4Dx Plus and modified Knott’s tests, 11 and 65 serum samples tested positive by the microtiter plate ELISA without and with preheating, respectively. Of these, nine samples were simultaneously positive by both the microtiter plate ELISA and two samples were positive only by the microtiter plate ELISA without preheating (Table 1).

Overall, the proportion of samples that tested positive for antigens was of 56.5% (205/363) and 79.6% (289/363) on the microtiter plate ELISA without and with preheating, respectively. The positive and the negative agreements between these methods were 70% (95% CI: 0.65–0.75) and 97% (95% CI: 0.91–1.00), respectively, with a statistically significant (McNemar test, *p* < 0.0001) difference between the methods.

The impact of immune complexes on antigen detection relative to each species of mfs identified by a modified Knott’s test was evaluated based on the number of samples found to be positive by the microtiter plate ELISA with preheating (n = 289). Samples were classified into three groups for the analysis. The first group included all samples positive for *D. immitis* mfs, including nine samples co-infected with *D. repens* (n = 159). The second group consisted of samples positive only for *D. repens* mfs (n = 15), whereas the third group included samples found to be negative by a modified Knott’s test (n = 115). The proportion of samples positive by the microtiter plate ELISA without preheating relative to those positive after heating was higher for dogs with *D. immitis* mfs (86%; 95% CI: 0.80–0.91) compared to samples positive only for *D. repens* mfs (47%; 95% CI: 0.25–0.70) or negative for mfs (51%; 95% CI 0.42–0.60). The latter two groups had an increased proportion of positive results on the microtiter plate ELISA with preheating (Table 2).

## 3. Discussion

The prevalence of *Dirofilaria* spp. in the studied sheltered dog population from a high-risk geographical area for HWD varies according to the diagnostic method employed, ranging from 44.4% (modified Knott’s method) to 79.6% (plate ELISA test with preheating), indicating how important it is the use of a multi-test diagnostic strategy for detecting positive dogs in a given population. All serum samples that tested positive using the SNAP 4Dx Plus test were positive by the microtiter plate ELISA reflecting the accuracy of the antigen detection of this POC test. A fairly similar proportion of infected dogs was obtained by combining the number of positive samples by the modified Knott’s test and the SNAP 4Dx Plus test with those analysed only by the microtiter plate ELISA without preheating (58.1% vs 56.5%, respectively). As in previous studies, the microtiter plate ELISA, regardless of the test used, provided a higher proportion of antigen-positive results than the POC ELISA test [20,24,25]. Nonetheless, the microtiter plate ELISA test with preheating detected the most positive test results (79.6%) in the studied dog population.

The difference in the proportion of positive results between the two microtiter plate ELISA methods is likely due to antigen–antibody immune complexes, which are known to interfere with the detection of HW antigen in immunoassays [21,22,26,27]. Antigen–antibody immune complexes occur when the dog’s antibodies bind to the carbohydrate epitopes of the HW antigens, making these regions unavailable for the antibodies used in the diagnostic assay [21]. As observed in this study, the negative agreement between the two microtiter plate ELISA tests was high (97%), indicating that samples negative for HW antigen after heating were also negative without heating. Contrarily, the low positive percent agreement (70%) reflects those samples that were only positive for antigen after heating, suggesting that this procedure acted by disrupting immune complexes and liberated the antigens, allowing them to be detected by the capture antibody present in the assay.

One concern that has been raised regarding the use of the ICD method is the potential for detecting similar carbohydrate antigens from other nematode parasites. Indeed, a previous study has shown that immunoassays for the detection of *D. immitis* antigen can detect excretory/secretory antigens obtained *in vitro* from other parasites, including *D. repens* [19]. Several different immunoassays demonstrated improved detection of *D. repens* antigen after heating [19,20,25]. However, in the current study, the modified Knott’s test identified both *D. immitis* and *D. repens* mfs in the examined dog blood samples, with the former infection predominating. This may be consistent with the number of occult *D. immitis* infections detected by the POC ELISA. Nevertheless, the true frequency of co-infections is difficult to determine, considering that the microtiter plate ELISA with preheating may have dissociated either *D. immitis* or *D. repens* antigen from immune complexes.

Given the relatively large proportion of samples that converted from antigen negative to positive after heating in the studied dog population, it was of interest to evaluate the test results based on the identification of the mfs by the modified Knott’s test. Based on the Knott’s results, the increase in the number of antigen-positive results after heating was greater in samples negative for mfs and in samples positive only for *D. repens* mfs as compared to those containing *D. immitis* mfs. The relative amounts of antigen and antibody in circulation determine the degree of immune complex formation and the residual amount of free antigen available for detection by serological tests. Several hypotheses for future studies could be suggested from these observations. First, patent *D. immitis* infections might be expected to have higher circulating antigen concentrations given that the antigen is shed from the uterus of the mature female parasite as the mfs are released [23]. On the other hand, *D. repens* infections are typically localized to the subcutaneous tissues and may release lower concentrations of antigen directly into circulation. From the results obtained in this study, it appears that amicrofilaremic dogs that were HW antigen positive would fall somewhere in between these two scenarios.

The modified Knott’s test detected fewer *D. immitis* infections than the microtiter plate ELISA antigen tests in this study. In particular, 30.3% of dogs negative for microfilaria using the Knott’s test in a previous study [10] reverted to a positive result using the plate ELISA test with preheating. This finding is also supported by previous studies [12,16]. Indeed, the absence of mfs in the canine blood may depend on several factors, such as the long prepatent period of the parasite (i.e., about 7 months) and the low mfs concentration in the samples [1,16,28,29]. Dogs may become amicrofilaremic after 12 months post-infection due to the development of an immune response or to female HW senility in the absence of reinfections [30]. In contrast, the highest level of mfs in the blood is detected between 7 and 12 months post-infection and during the warmest seasons (i.e., between June and September), with circadian peaks occurring from 4:00 a.m. to 10:00 p.m. [30,31]. On the other hand, although the modified Knott’s method allows the morphological identification of the mfs, no other filarial species were herein detected, probably due to the low pressure of proper vectors in the given environment.

This study has some limitations. Only two dog shelters were herein investigated, which could lead to sampling bias. In addition, the SNAP 4Dx Plus test was only performed on samples scored negative by the modified Knott’s test. Thus, a full comparison between the modified Knott’s test, the SNAP 4Dx Plus test and the microtiter plate ELISA methods was not possible. Although an increase in the number of positive samples after heating was more evident for *D. repens*-positive samples, the sample size for this conclusion was small.

The accuracy of the diagnostic methods for the detection of HW infection in dogs is essential for veterinary practitioners to decide which is the most suitable strategy for each dog, that is, curative or preventive treatments for positive and negative dogs, respectively [10,23]. Furthermore, chemoprophylaxis should be addressed in dog shelters, where dogs are confined and oftentimes much more exposed to mosquito vectors in the outside environment, as compared to single privately owned dogs. These results also suggest that veterinary practitioners dealing with dogs without clinical signs suggestive of HWD should interpret the results of qualitative serological tests with caution and, preferably, in combination with a modified Knott’s test for circulating mfs. Both results should always be interpreted in conjunction with detailed clinical and anamnestic data. Considering the data above, the combination of more than one diagnostic tool is recommended to increase the probability of finding true positive dogs. Although the microtiter plate ELISA with preheating has been shown to detect more HW-antigen-positive dogs [26,27], this method is presently not recommended for routine HW screening (American Heartworm Society guidelines). Indeed, dogs receiving annual veterinary care and HW prevention were not found to have a high likelihood of false-negative HW antigen test results due to immune complexing [32]. Furthermore, the detection of HW antigens using the SNAP 4Dx Plus test remains one of the most common and reliable techniques to be used for a rapid POC diagnosis in veterinary clinics as well as in field studies [20,33]. From a public health perspective, these data highlight that the use of the modified Knott’s test alone may result in many false-negative dogs, serving as a hidden source of infection to mosquito vectors, thus representing a risk for other dogs and humans sharing the same environment.

## 4. Materials and Methods

### 4.1. Study Design

Blood and serum samples (n = 363) used herein were collected during a previous study on *Dirofilaria* spp. infection in sheltered dogs [10]. All dogs were housed in two shelters in Southern Italy (40.608705N, 17.994495E, site 1; 40.419326N, 18.165582E, site 2), where 44.2% and 7% were infected by *D. immitis* and *D. repens*, respectively, as determined by the modified Knott’s test [10]. The animals were handled and sampled following the approval of the Ethical Committee of the Department of Veterinary Medicine of the University of Bari, Italy (Prot. Uniba 8/19).

### 4.2. Diagnostic Procedures

Two HW antigen detection tests were used in this study: the rapid SNAP 4Dx Plus Test (IDEXX Laboratories, Inc., Westbrook, ME, USA) and the microtiter plate ELISA (i.e., Heartworm Antigen by ELISA, IDEXX Laboratories, Inc., Westbrook, ME, USA) with and without a heat pretreatment method for ICD of the serum sample. The microtiter plate ELISA, which is only available through the IDEXX Reference Laboratory, was performed as previously described [23]. Serum samples (100 µL) were added to each well of the microtiter plate, coated with the capture antibody and incubated for 30 min at room temperature. Each well was rinsed five times with wash solution and then 100 µL of a horseradish peroxidase-conjugated antibody solution was added to the well and incubated for 30 min at room temperature. The wells were washed and 50 µL of 3,3′,5,5′-tetramethylbenzidine (TMB) substrate solution was added and incubated for 10 min at room temperature. Following the addition of a 0.1% sodium dodecyl sulfate stop solution, the absorbance was measured at 650 nm. A positive result was recorded if the absorbance of the sample exceeded that of the negative control by an optical density (OD) of 0.05. For the microtiter plate ELISA with preheating, the serum sample was mixed with an equal volume of 0.1 M EDTA (pH 7.5) and the mixture was heated at 100 °C for 5 min [23]. Following centrifugation at 16,000× *g* for 5 min, the supernatant (100 µL) was transferred to the microtiter plate and the ELISA performed as described above. The microtiter plate ELISA for the heartworm antigen was used to test all samples. The SNAP 4Dx Plus Test, a bi-directional flow ELISA test designed for POC testing, was performed according to the manufacturer’s instructions for any sample that scored negative on the modified Knott’s test. Before testing, each serum sample was thawed at room temperature and then vortexed.

### 4.3. Data Handling and Statistical Analysis

In the absence of a gold standard (i.e., necropsy), percent positive and negative agreements were determined for the microtiter plate ELISA, both with and without preheating, by calculating the proportion of samples found to be positive by both methods over the total number of positives by the ICD method. Likewise, percent negative agreement was the proportion of negative samples found by both methods over the total number of negative samples by the ICD method. Concordance between ELISA with and without preheating was evaluated by McNemar’s test for paired data. Statistical significance was defined as a p-value < 0.05. Exact binomial methods were used to calculate the 95% confidence intervals for both percent agreements. All analyses were performed using R (version 3.6.1). Visualization of data sets and test results employed Euler diagrams (created in Microsoft PowerPoint, 2016).

## Figures and Tables

**Table 1:** Modified Knott’s test microfilaria negative samples (n = 184) tested for *D. immitis* antigen by SNAP 4Dx Plus and microtiter plate ELISA with and without preheating.

Knott’s Test Negative Samples (n = 184)	SNAP 4Dx Plus	HW Ag ELISA without Preheating	HW Ag ELISA with Preheating
50	Positive	Positive	Positive
9	Negative	Positive	Positive
56	Negative	Negative	Positive
2	Negative	Positive	Negative
67	Negative	Negative	Negative

**Table 2:** Comparison of heartworm antigen detection by microtiter plate ELISA without and with preheating relative to Knott’s test results for those samples that tested antigen positive after preheating.

Modified Knott’s Method	HW Ag ELISA without Preheating	HW Ag ELISA with Preheating	Increase in Positive Samples with vs. without Preheating (%)
*Dirofilaria immitis* and co-infected ^a^	137	159	16%
*Dirofilaria repens* only	7	15	114%
No microfilaria observed	59	115	95%
**Total**	**205**	**289**	**42%**

^a^ This includes nine dogs positive for both *D. immitis* and *D. repens*.
